# Dissolved oxygen isotope modelling refines metabolic state estimates of stream ecosystems with different land use background

**DOI:** 10.1038/s41598-022-13219-9

**Published:** 2022-06-17

**Authors:** David R. Piatka, Jason J. Venkiteswaran, Bhumika Uniyal, Robin Kaule, Benjamin Gilfedder, Johannes A. C. Barth

**Affiliations:** 1 Department of Geography and Geosciences, GeoZentrum Nordbayern, Friedrich-Alexander-Universität Erlangen-Nürnberg (FAU), Schlossgarten 5, 91054 Erlangen, Germany; 2 Department of Geography and Environmental Studies, Wilfrid Laurier University, 75 University Avenue West, Waterloo, ON N2L 3C5 Canada; 3 Professorship of Ecological Services, Bayreuth Center of Ecology and Environmental Research (BayCEER), University of Bayreuth, Universitaetsstr. 30, 95447 Bayreuth, Germany; 4 Limnological Research Station, BayCEER, Department of Hydrology, University of Bayreuth, 95440 Bayreuth, Germany; 5 Present Address: Karlsruhe Institute of Technology, Institute of Meteorology and Climate Research (IMK-IFU), Kreuzeckbahnstr. 19, 82467 Garmisch-Partenkirchen, Germany

**Keywords:** Biogeochemistry, Environmental sciences

## Abstract

Dissolved oxygen (DO) is crucial for aerobic life in streams and rivers and mostly depends on photosynthesis (P), ecosystem respiration (R) and atmospheric gas exchange (G). However, climate and land use changes progressively disrupt metabolic balances in natural streams as sensitive reflectors of their catchments. Comprehensive methods for mapping fundamental ecosystem services become increasingly important in a rapidly changing environment. In this work we tested DO and its stable isotope (^18^O/^16^O) ratios as novel tools for the status of stream ecosystems. For this purpose, six diel sampling campaigns were performed at three low-order and mid-latitude European streams with different land use patterns. Modelling of diel DO and its stable isotopes combined with land use analyses showed lowest P rates at forested sites, with a minimum of 17.9 mg m^−2^ h^−1^. Due to high R rates between 230 and 341 mg m^−2^ h^−1^ five out of six study sites showed a general heterotrophic state with P:R:G ratios between 0.1:1.1:1 and 1:1.9:1. Only one site with agricultural and urban influences showed a high P rate of 417 mg m^−2^ h^−1^ with a P:R:G ratio of 1.9:1.5:1. Between all sites gross G rates varied between 148 and 298 mg m^−2^ h^−1^. In general, metabolic rates depend on the distance of sampling locations to river sources, light availability, nutrient concentrations and possible exchanges with groundwater. The presented modelling approach introduces a new and powerful tool to study effects of land use on stream health. Such approaches should be integrated into future ecological monitoring.

## Introduction

Streams and rivers are among the most important indicators of the environmental states of our continents^1–4^. They are also the most important transporters of material from continents to oceans and as the lowest lineaments in landscapes they integrate water and its dissolved constituents from catchments^5–7^. In addition, rivers, streams, and their riparian ecosystems, including the hyporheic zone (HZ), are important reflectors of continental carbon and oxygen cycles that are currently undergoing drastic changes due to rapid environmental changes of climate and land use^8–12,13^.

Dissolved oxygen (DO) is pivotal for the survival of aquatic aerobic life. It is also necessary for nutrient cycling and plays a central role in organic carbon oxidation^14,15^. The majority of studies on rivers and streams measure DO routinely and often with a high resolution^16–18^. However, DO sources and sinks often remain unknown. Physical processes that control DO concentrations include gas exchange (G) with the atmosphere. Biological processes include aquatic ecosystem metabolism with respiration (R) as a DO sink and photosynthesis (P) as a source. These three processes are key drivers of the DO pool on hourly to seasonal timescales^19^. G is independent of the time of day and always acts to drive DO concentrations towards atmospheric equilibrium. During daytime P by autotrophs typically increase DO and can lead to oversaturation in the water column. On the other hand, R by heterotrophs can cause undersaturation, especially when P is low or absent at night. Such DO losses become enhanced when G rates are low.

An understanding of these processes is essential for aquatic ecosystems and recent analyses of the GLobal RIver CHemistry database (GLORICH) suggested increasingly heterotrophic states of rivers, that may further deteriorate due to future global warming^2,20^. Such trends underline the importance of establishing new and integral tools of DO source and sink quantifications that can help to quantify metabolic state and ecological functioning of aquatic environments. Those tools can help to characterize system functioning and may contribute to early recognition of shifts in DO source and sink terms. Such evaluations may also enable mitigation of deleterious consequences for stream ecosystems and their biota^19,21–24^.

**Figure 1:**
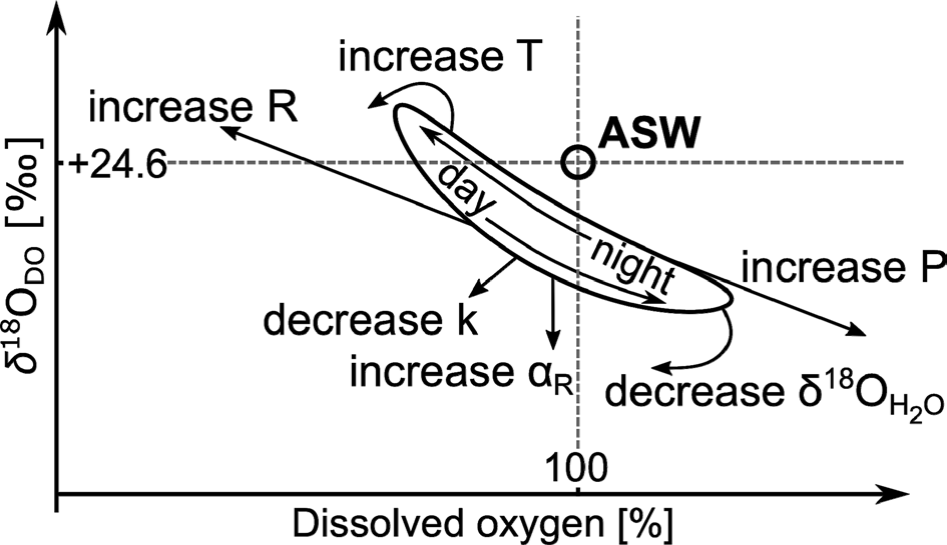
Schematic illustration of effects on dissolved oxygen (DO) and its stable DO isotopes (*δ*^18^O_DO_) by photosynthesis (P) and ecosystem respiration (R) with the respiration isotope fractionation factor (α_R_), the gas exchange coefficient (k), stable water isotopes (*δ*^18^O_H2O_) and water temperature (T) after Venkiteswaran et al.^27^. Horizontal and vertical dashed lines and the empty circle represent air saturated water (ASW) with DO saturation at 100% and *δ*^18^O_DO_ at + 24.6‰. The oval line represents a typical hysteresis curve during the course of a diel cycle.

In earlier aquatic metabolism studies, P:R ratios were exclusively used to describe metabolic states of stream ecosystems^25,26^. However, more recent studies questioned the use of P:R ratios alone and as an improved measure encouraged the inclusion of G as an important third parameter to formulate P:R:G ratios^24,27,28^. Additionally, newer and more complex studies have shown the usefulness of combining DO measurements with their corresponding stable isotopes (*δ*^18^O_DO_) as a complementary parameter^29,30^. In such approaches measurements of *δ*^18^O_DO_ values add additional information to the metabolic assessment of the stream ecosystem^31–33^. For example, when observed DO saturations are at 100% they might indicate equilibrium with the atmosphere. However, corresponding *δ*^18^O_DO_ values might still be lower than those expected for air saturated water (ASW) with a value of + 24.6‰^31,34^. This would be the case, when a residual photosynthetic signal still influences the DO pool^24,27,28^. As demonstrated by Venkiteswaran et al.^27^ and based on data from Wilcock et al.^35^, shapes of diel DO and *δ*^18^O_DO_ curves are distinct. This dual approach provided well-constrained respiration isotope fractionation factors (α_R_) and respiration rates based on diel *δ*^18^O_DO_ curves. Hence, combining independent measurements of both parameters allows to quantify diel DO patterns from two different perspectives and improves model output of P, R and G rates with smaller error ranges. Key parameters that control metabolic rates include water temperature, hydrological conditions and light availability abundance of carbon and nutrients^19,20,36–38^. Further specific parameters that influence both DO saturations and their corresponding *δ*^18^O_DO_ values include the coefficient for atmospheric gas exchange (k), α_R_, and the stable isotope value of the source water *δ*^18^*O*_H2O_. Figure 1 summarizes interactions of diel curves of DO and *δ*^18^O_DO_ with these environmental parameters.

In this study, we applied a new dynamic DO stable isotope model on P, R and G, that is abbreviated as PoRGy. It was first described by Venkiteswaran et al.^24^. The aim of this study was to test a new DO and *δ*^18^O_DO_ data set with the PoRGy model on three contrasting mid-latitude European streams. The streams of investigation are located in the same climatic zone within Germany. However, their catchments have varying background geologies and land use patterns. A related goal was to identify ratios of P:R:G in each stream in an upstream and downstream section. The resulting data set enabled comparisons of these ratios within each stream. Such tests can help to judge if differences between or within streams are more pronounced. Furthermore, we aimed to assess how different land use patterns within each catchment and associated different nutrient and light availabilities may affect stream metabolism. The study is timely because it presents a new and integral technique to evaluate stream metabolism in an efficient manner by the combined application of DO concentrations and stable isotopes. This approach yields well-constrained P, R and G rates for comparison of stream ecosystems. With rapidly changing environmental conditions in streams and rivers such tools are essential for early recognition of environmental change streams and their catchments. This study also is the first application of this technique in Europe that combines diel DO isotopes with data on land use patterns.

## Methods

### Study sites

**Figure 2:**
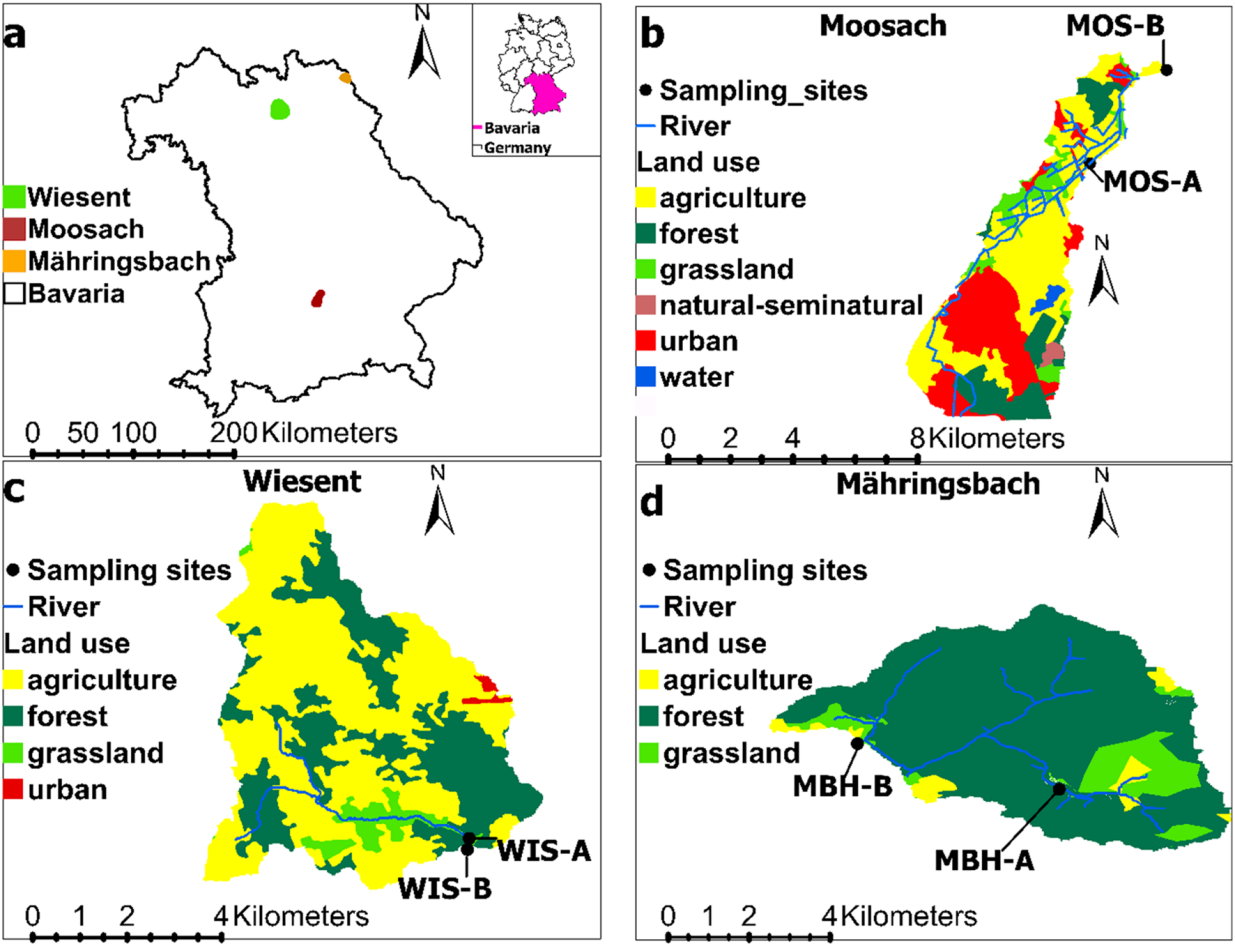
Studied streams with land use characteristics upstream of sampling sites. (**a**) Overview map of Bavaria with locations of the three streams Mähringsbach (MBH) in orange, Wiesent (WIS) in green and Moosach (MOS) in red with their respective catchments. Subfigures (**b**–**d**) mark detailed land use patterns of MOS, WIS and MBH catchment sections, respectively, while study sites A and B represent upstream and downstream locations. The map was created with the geographic information system (GIS) software ArcGIS Pro, version 2.7.2 (https://www.esri.com/en-us/arcgis/products/arcgis-pro/overview).

The three streams Mähringsbach (MBH), Wiesent (WIS) and Moosach (MOS) each with one selected upstream (A) and downstream (B) study site are located in Bavaria, Southern Germany, with different geological and land use backgrounds (Table 1, Fig. 2a–d).

**Table 1:** Overview of sampled streams and study sites with respective GPS coordinates.

Stream	Sampling site	Latitude (degree North)	Longitude (degree East)
Mähringsbach	MBH-A	50.248084	12.095404
Mähringsbach	MBH-B	50.256801	12.052282
Wiesent	WIS-A	49.973019	11.190348
Wiesent	WIS-B	49.970896	11.189733
Moosach	MOS-A	48.323114	11.622429
Moosach	MOS-B	48.349319	11.656940

The MBH stream originates in the Czech Republic, where it is known as “Üjezdsky potok”. The selected study site MBH-A is located within the forest “Rehauer Forst” and in the vicinity to the source region. The second study site of this stream system (MBH-B) was approximately 5 km downstream from MBH-A (Fig. 2d).

The WIS stream is located in the Franconian Alb, South Germany. It is dominated by karst lithology mostly of calcites and dolomites. The two selected study sites WIS-A and WIS-B were selected at distances of 2.9 and 3.3 km from the source, respectively (Fig. 2c).

The MOS stream is a tributary of the river Isar and flows in the Alpine foothills in Southern Bavaria. The study sites MOS-A and MOS-B were chosen between the cities of Munich and Freising. They are approximately 5 km apart from each other (Fig. 2b).

### Field methods

In total six diel sampling campaigns of at least 28 h were carried out. In order to outline the strongest diel differences between metabolic rates all sampling campaigns were carried out in summer. The upstream and downstream study site of each stream was sampled in 2-h time intervals. At MBH-A the end of the sampling was extended until 4 pm due to loss of samples that were collected between 10:00 and 12:00 a.m.

All water samples were collected in the middle of each stream by syringe at 20 cm depth below the water surface, if possible. Before sample collection syringes were rinsed three times with sample water. Samples were then filtered through 0.45-µm disk filter (Minisart HighFlow PES, Sartorius AG, Germany). For oxygen and hydrogen stable isotopes of water (*δ*^2^H_H2O_ and *δ*^18^O_H2O_) samples were collected in 12-mL glass bottles. Samples for the ^18^O/^16^O ratios of dissolved oxygen (expressed as *δ*^18^O_DO_) were collected in 12-mL Labco Exetainers™ (Labco Ltd. Lampeter, U.K). These were pre-poisoned with 20 µL of a saturated HgCl_2_ solution in order to avoid secondary biological activity after sampling. The vials were completely filled and immediately capped using screw caps with a butyl rubber septum. Previous laboratory-internal tests showed negligible contamination by atmospheric O_2_ when applying this sampling method.

Measurements of water temperature and DO were conducted in the field with a multi-parameter instrument (Multi 3620 IDS/3430 by WTW GmbH, Weilheim, Germany). All probes were calibrated at least once per day. One σ-repeat measurements of temperature was better than ± 0.1 °C and ± 2% for DO.

Discharge measurements are an important input to the PoRGy model and were determined with an electromagnetic currentmeter (SEBA Hydrometrie GmbH FlowSens) that was placed along a transect across the stream at two different depths (close to the surface and close to the bottom) with the 2-point-measurement method of Kreps^39^.

### Laboratory methods, *stable isotope measurements*

Water samples were analyzed for *δ*^18^O of DO with a modified method by Barth et al.^40^. The method couples an automated equilibration unit (Gasbench II) to a Delta V Advantage isotope ratio mass spectrometer (ThermoFisher Scientific, Bremen, Germany). The isolation of DO into a headspace relies on a helium extraction technique by Kampbell et al.^41^ and Wassenaar and Koehler^42^. Prior to analyses, headspaces were automatically generated in each water-filled vial on the Gasbench II with an autosampler that was equipped with a double-hole needle. After headspace generation samples were placed for 30 min on a horizontal shaker that moved at a rate of 250 strokes per minute to mobilize all DO into the headspace. Subsequently, samples were placed back on the Gasbench II autosampler after a switchover to connect with the isotope ratio mass spectrometer. The headspace was then mobilized in a helium stream via another dry double-hole needle on the autosampler. The O_2_ was separated by a CP-Molsieve 5 Å capillary column (25 m length Å ~ 0.53 mm OD Å ~ 0.05 mm ID; Agilent, Santa Clara, CA, USA). The purified O_2_ was then transferred by continuous flow to the mass spectrometer. Laboratory air was used as an internal standard with a known value of + 23.88‰^43^. Further details of the method are available in Köhler et al.^44^.

Water samples were analyzed for their *δ*^18^O_H2O_ values by isotope ratio infrared spectroscopy (IRIS) that operates based on wavelength-scanned cavity ring-down time measurements (L2120-i, Picarro Inc., Santa Clara, CA, USA). Each sample was measured by nine injections of which the first three injections were discarded to exclude memory effects.

All water isotope measurements were normalized against two international reference materials named Vienna Standard Mean Ocean Water (VSMOW) and Standard Light Antarctic Precipitation (SLAP). This two-point calibration was controlled by a third laboratory reference water that was calibrated directly against VSMOW and SLAP.

Isotope results were reported in the standard delta notation in per mil (‰) versus VSMOW according to

1 $${\delta }^{18}\mathrm{O}=\frac{{}{}^{18/16}{R}_{s}}{{}{}^{18/16}{R}_{r} }-1$$

where ^18/16^R_s_ is the oxygen isotope ratio of heavy to light isotopes in the sample and ^18/16^R_r_ is the ratio in the standard (VSMOW, 0.0020052 ^45^). All values were then multiplied by 1000 in order to convert them to permille (‰). Repeat measurements of field standards revealed a standard deviation of ± 0.2‰ (± 1 σ) for both, *δ*^18^O_DO_ and *δ*^18^O_H2O_. All samples were measured in triplicates and reported values are averages.

### PoRGy model

The PoRGy model enabled evaluations of metabolic rates and G via diel changes in DO concentration and *δ*^18^O_DO_ values at each location^24,27^. This approach resembles curve-fitting procedures for diel DO concentrations measurements, but adds *δ*^18^O_DO_ as another parameter to constrain rates. P rates were modelled by calculating incident light via known latitude, longitude, and day of year to a maximum P rate. Photosynthetic O_2_ has the same *δ*^18^O_DO_ value as the source *δ*^18^O_H2O_ without fractionation (α_P_ = 1.000). With this, DO concentrations should increase when *δ*^18^O_DO_ value decrease^46,47^. R consumes DO with in dependence of temperature. This process was modelled with a modified Arrhenius equation and a well-described range of possible α_R_ values. According to this principle, DO concentrations decrease when the *δ*^18^O_DO_ value increase^25,48–52^. Net gas exchange between water and atmosphere is controlled by the level of saturation, that in turn depends on water temperature and the atmospheric equilibrium constant k^53^. A comparatively small equilibrium isotope fractionation enrichment of atmospheric O_2_  with a value of + 23.88‰ towards DO involves a temperature-dependent enrichment of about + 0.7‰^43,54^.

Data for each site were modelled with boundary conditions placed on each of the five parameters described above (P, R, k, α_R_, and *δ*^18^O_H2O_). Metabolic rates were allowed to vary by two orders of magnitude, k was allowed to vary by a range of 50% wider than values calculated from stream velocity and depth^55–58^, α_R_ was allowed to vary between 0.975 and 1.000, and *δ*^18^O_H2O_ was allowed to vary by 0.5‰ around its measured value. The Matlab (The MathWorks Inc., version R2021a Natick, Massachusetts; http://www.mathworks.com) implementation of PoRGy selected initial values for these variables from within these constraints. The fminsearch function then altered these constrained variables to find a minimum sum-of-squared errors between field data and model output. The r^2^ values for field data and model output for both DO and *δ*^18^O_DO_ values are a measure for the quality of fit. This approach is similar to the one described in Wassenaar et al.^28^.

In addition to the PoRGy model with the application of DO saturations and stable isotopes, we also tested the model performance based on DO concentrations alone in order to assess benefits of the combined approach.

### GIS analyses

For the analyses of the stream catchments the geographic information system (GIS) software ArcGIS Pro (https://www.esri.com/en-us/arcgis/products/arcgis-pro/overview), version 2.7.2, was applied. A 30 m digital elevation map of Bavaria (https://dwtkns.com/srtm30m/), the stream network from Bayerisches Landesamt für Umwelt (LFU) and the stream gauging locations (Table 1) were used as inputs for creating the catchments of the three watersheds in this study. Catchments were delineated using the ArcSWAT tool (https://www.arcgis.com/index.htm). Land use patterns were extracted using the clip tool in ArcGIS from the CORINE land cover map for 2018 (https://land.copernicus.eu/pan-european/corine-land-cover).

## Results

A detailed overview of measured DO concentrations and saturations, water temperatures, nutrient concentrations, and *δ*^18^O_DO_ and stable isotopes of water (*δ*^18^O_H2O_) at the streams MBH, WIS and MOS is provided in the supplementary material (Table S1).

### Water depths, flow velocities and water temperatures

Measurements took place during low to medium water discharge at all sites^59–61^. Input values for water depths, flow velocities and water temperatures varied between the streams MBH, WIS and MOS, and upstream and downstream locations (Table 2). Water depths and flow velocities were averaged over the river width from four sampling locations during the sampling period. Flow regimes were stable within the boundaries of measurement uncertainties. The maximum water depth was 0.46 m and the minimum value 0.05 m. Flow velocities ranged between 0.03 and 0.72 m s^−1^, while water temperatures ranged between 10.5 and 22.6 °C.

**Table 2:** Overview of water depths, mean flow velocities and water temperature minima, maxima and averages of the upstream (A) and downstream (B) sampling sites at the streams Mähringsbach (MBH), Wiesent (WIS) and Moosach (MOS).

Date	Sample ID	Water depth (m)	Mean flow velocity (m s^−1^)	Min. water temperature (°C)	Max. water temperature (°C)	Mean water temperature (°C)
24./25.07.2019	MBH-A	0.05	0.17	13.1	20.4	16.7
24./25.07.2019	MBH-B	0.08	0.03	16.8	22.6	19.4
06./07.08.2019	WIS-A	0.15	0.36	11.8	15.1	13.3
06./07.08.2019	WIS-B	0.40	0.26	10.5	12.5	11.4
16./17.07.2019	MOS-A	0.50	0.17	12.9	16.5	14.6
16./17.07.2019	MOS-B	0.46	0.72	14.3	16.9	15.4

### DO and *δ*^18^O_DO_

**Figure 3:**
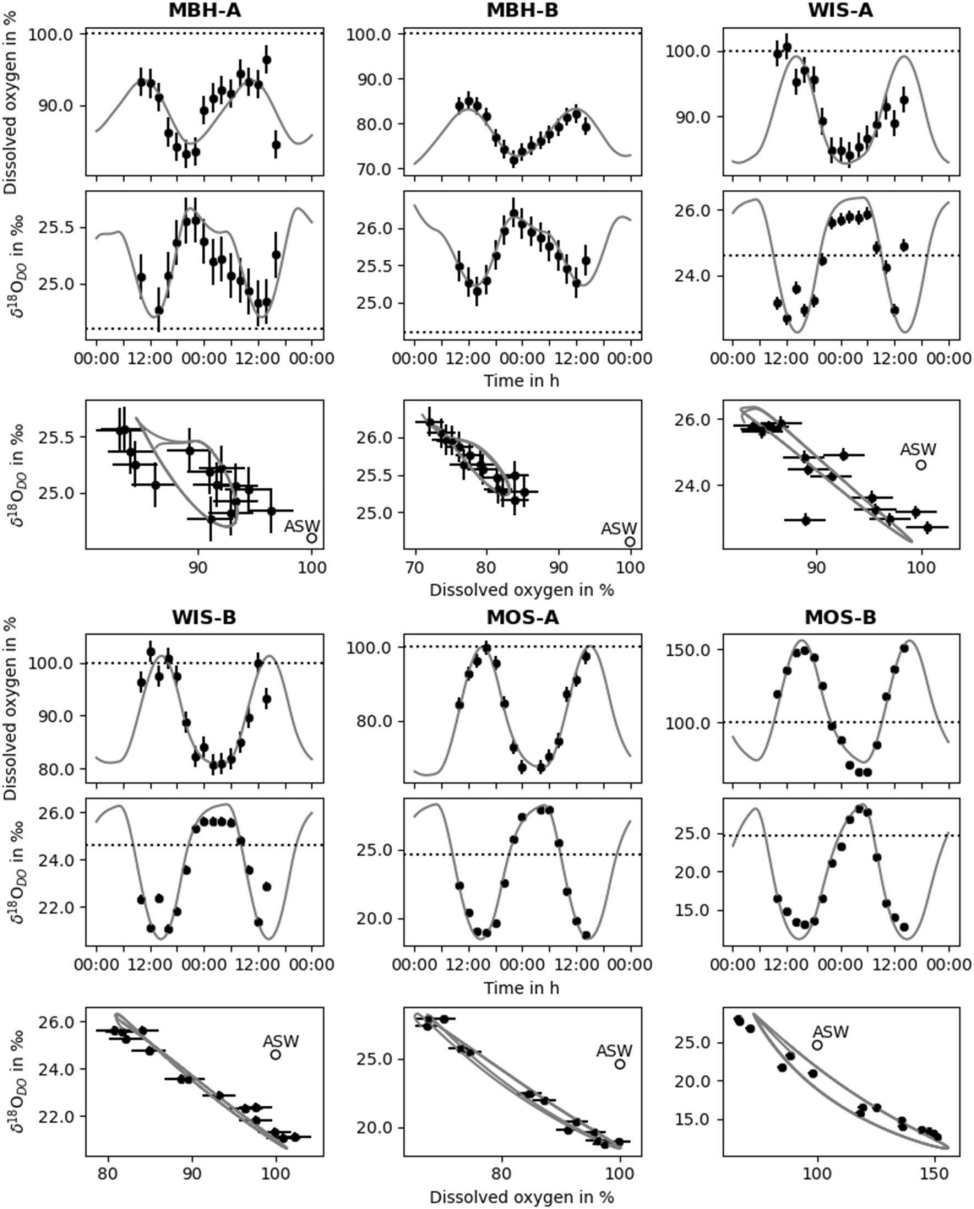
Overview of day–night field data (black dots) and PoRGy model results (grey lines) of dissolved oxygen (DO) saturation and stable DO isotopes (*δ*^18^O_DO_) at the study sites A (upstream) and B (downstream) of the streams Mähringsbach (MBH), Wiesent (WIS) and Moosach (MOS). Horizontal dashed lines and the empty circle represent air saturated water (ASW) with DO saturation of 100% and a *δ*^18^O_DO_ value of + 24.6‰. Standard errors for DO saturation and *δ*^18^O_DO_ are 2% and 0.2‰, respectively.

All sampling sites revealed major diel changes in DO concentrations, saturations and corresponding *δ*^18^O_DO_ values. These variations had site-specific ranges, shapes, and magnitudes within and especially between the streams (Table 3, Fig. 3). All *δ*^18^O_H2O_ values (as the source for newly photosynthetically produced DO) varied within a small range for all sites. This homogeneous input signal enables a direct comparison of the *δ*^18^O_DO_ values (supplementary material, Table S1). Moreover, DO saturations of all study sites showed strong negative relationships with *δ*^18^O_DO_ (r^2^ = 0.86, p < 0.001). Times of minima of DO saturations did often not precisely match with times of maxima *δ*^18^O_DO_.

**Table 3:** Summary of measured minima and maxima of DO concentrations and saturations, and stable DO isotopes (*δ*^18^O_DO_) values at Mähringsbach (MBH), Wiesent (WIS) and Moosach (MOS) with respective upstream (A) and downstream (B) sites.

Date	Study site	Min. DO (mg L^−1^)	Max DO (mg L^−1^)	Min DO (%)	Max DO (%)	Min. *δ*^18^O_DO_ (‰)	Max. *δ*^18^O_DO_ (‰)
24./25.07.2019	MBH-A	7.2	9.2	83.1	96.4	24.8	25.6
24./25.07.2019	MBH-B	6.0	7.7	72.0	85.2	25.2	26.2
06./07.08.2019	WIS-A	8.3	10.2	84.0	100.6	22.7	25.9
06./07.08.2019	WIS-B	8.4	10.6	80.7	102.3	21.1	25.6
16./17.07.2019	MOS-A	6.5	9.3	67.4	99.7	18.8	27.9
16./17.07.2019	MOS-B	6.4	14.1	65.9	151.1	12.7	28.1

The highest measured DO saturations and lowest measured *δ*^18^O_DO_ occurred slightly before or after the solar noon period between 12:00 and 16:00 h (Fig. 3). However, due to the chosen sampling interval of 2 h, actual peaks may have been missed. In contrast, the lowest DO saturations were found between 20:00 and 22:00 h with the highest *δ*^18^O_DO_ values at 22:00 h in the MBH stream. In the other two streams DO saturation minima occurred between 00:00 and 04:00 h with corresponding *δ*^18^O_DO_ maxima between 04:00 and 06:00 h.

During daytime all sites showed increases in DO saturations. However, only site MOS-B reached clearly oversaturated values, whereas the other sites remained undersaturated or close to atmospheric equilibrium during the entire day. In contrast, at night all streams became DO undersaturated. Among the study sites, a night plateau in DO and *δ*^18^O_DO_ values was best visible in the WIS and MOS streams (Fig. 3).

The MBH stream maintained DO undersaturation at both study sites and revealed the smallest range of DO saturation and *δ*^18^O_DO_. DO saturations at the sites MBH-A and MBH-B followed a more irregular day–night cycle with a range from 83.1 to 96.4% (at 20:00 and 14:00 h) and 72.0 to 85.2% (at 22:00 and 12:00 h), respectively (Table 3). At these two sites, corresponding *δ*^18^O_DO_ values also permanently remained above atmospheric equilibrium (i.e., >  + 24.6‰) with values ranging between + 24.8 and + 25.6‰ at MBH-A and between + 25.2 and + 26.2‰ at MBH-B.

Daytime variations of DO were more pronounced in the WIS stream with DO maxima of 100.6 and 102.3% (both at 12:00 h) and DO minima of 84.0 and 80.7% (both 02:00 h) at WIS-A and WIS-B, respectively. Corresponding *δ*^18^O_DO_ values followed opposite trends but remained at values below atmospheric equilibrium during the day (WIS-A: + 22.7‰; WIS-B: + 21.1‰) and above atmospheric equilibrium during the night (WIS-A: + 25.9‰; WIS-B: + 25.6‰). At the end of the diel sampling interval these DO and *δ*^18^O_DO_ curves showed unexpected values for daytime with a shift to lower DO and higher *δ*^18^O_DO_ values that indicate dominance of R.

In the MOS stream the two selected study sites showed the most pronounced diel patterns. With a DO saturation mean of 84.4%, MOS-A was predominantly undersaturated and only approached atmospheric equilibrium with a maximum of 99.7% at 16:00 h (*δ*^18^O_DO_; 18.8‰ at 14:00 h). After this peak DO values decreased and reached a minimum of 67.4% at 00:00 h with a corresponding *δ*^18^O_DO_ value of + 27.9‰ at 04:00. In contrast, study site MOS-B revealed a much stronger diurnal range with oversaturated DO values of up to 151.2% and low corresponding *δ*^18^O_DO_ values of + 12.7‰ at 14:00 h. The same site showed a strong undersaturation at night with 65.9% and a corresponding *δ*^18^O_DO_ of + 28.1‰ at 04:00 h. With an average DO saturation of 113.6% during the whole diel sampling campaign, this site was most oxygenated.

### DO and *δ*^18^O_DO_ modelling results

All modelled DO and δ^18^O_DO_ diel curves matched well with the measured data (Fig. 3). The modelling approach was to adjust P, R, k, α_R_, and δ^18^O_H2O_ to find a best-fit solution. This yielded average midnight-to-midnight P, R, and G rates from the modelled diel curves. Generally, modelled diel curves followed the general day-night course of the data and outlined timing of DO and δ^18^O_DO_ minima and maxima as well as nighttime plateaus (Fig. 3). The goodness of fit (r^2^) was best for the MOS stream with values ranging between 0.97 and 0.99 and varied between 0.63 and 0.89 at the other sites. Moreover, the modelled *δ*^18^O_DO_ curves always showed a better fit with measured data than their corresponding DO curves.

When running the model based only on DO saturations, model fits were similar compared to combined DO and *δ*^18^O_DO_ modelling**.** Only site MBH-A showed a lower r^2^ of 0.52 (supplementary material, Table S2)**.**

### Metabolic rates and ratios

For a better comparison of the different streams and study sites, the modelled midnight-to-midnight P, R and gross G rates (G*) and ratios are valuable indicators of the metabolic states of the three sites.

Lowest P rates were found at the MBH stream with a value of 18 mg m^−2^ h^−1^ at MBH-B, while the highest P rates were found at the MOS stream with a value of 417 mg m^−2^ h^−1^ at MOS-B (Table 4). Minimum and maximum respiration rates were detected at the same sites with an R rate of 230 mg m^−2^ h^−1^ at MBH-B and 341 mg m^−2^ h^−1^ at MOS-B. Modelled G rates ranged between 148 mg m^−2^ h^−1^ at WIS-A and 298 mg m^−2^ h^−1^ at MBH-A.

**Table 4:** Overview of modelled midnight-to-midnight photosynthesis (P), ecosystem respiration (R) and gross gas exchange (G*) rates and ratios, and gas exchange (G) coefficients (k) at the streams Mähringsbach (MBH), Wiesent (WIS) and Moosach (MOS) with respective upstream (A) and downstream (B) sites.

Study site	Midnight-to-midnight						
	P rate (mg m^−2^ h^−1^)	R rate (mg m^−2^ h^−1^)	G* rate (mg m^−2^ h^−1^)	P:R ratio	P:G ratio	P:R:G ratio	k (m h^−1^)
MBH-A	39	340	298	0.1	0.1	0.1:1.1:1	0.30
MBH-B	18	230	214	0.1	0.1	0.1:1.1:1	0.11
WIS-A	89	238	148	0.4	0.6	0.6:1.6:1	0.14
WIS-B	128	284	157	0.5	0.8	0.8:1.8:1	0.14
MOS-A	163	327	171	0.5	1.0	1:1.9:1	0.09
MOS-B	417	341	221	1.2	1.9	1.9:1.5:1	0.09

The relative importance of metabolic processes and gas exchange is best reflected by P:R:G ratios (Table 4). The low primary productivity in the MBH stream compared to R and G yielded P:R:G ratios of 0.1:1.1:1 at MBH-A and MBH-B. At the WIS stream R was the main driver of the DO cycle with P:R:G ratios of 0.6:1.6:1 at WIS-A and 0.8:1.8:1 at WIS-B. Also, site MOS-A was dominated by R with a ratio of 1:1.9:1. In contrast, at study site MOS-B active photosynthesis (P) represented the highest DO fluxes on a diel basis. It was the only study site, where P exceeded R and G rates with a ratio of 1.9:1.5:1.

In contrast, the PoRGy model based solely on DO saturations yielded generally overestimated P, R and G rates. This effect was best visible at the low productivity sites MBH-A and B, with around 5 to 21 higher P rates and also significantly higher R and G rates (supplementary material, Table S2). This comparison with a single parameter approach of DO concentrations shows that the combined application of DO saturations and stable isotopes was much better in constraining P, R and G rates.

### Ecosystem respiration and fractionation factor α_R_

In order to optimize the model to the data, the fractionation factor for respiration α_R_ was allowed to vary within the range of known ecosystem respiration fractionation factors between 0.975 and 1.000. With this approach it was solved for best-fit combination of model input parameters^25,49,62^. The modelled α_R_ values ranged between 0.983 and 0.990. On average α_R_ values were slightly higher in the MBH stream (MBH-A with 0.989 and MBH-B with 0.990) than the WIS (WIS-A with 0.986 and WIS-B with 0.988) and MOS (MOS-A with 0.986 and MOS-B with 0.983).

### Nutrients

Phosphate values were mainly close to zero or below limits of quantification (LOQ = 0.001 mmol L^−1^), whereas average nitrate concentrations were low at the MBH stream with 0.07 (± 0.01; standard deviation) mmol L^−1^ at MBH-A and up to 0.09 (± 0.07) mmol L^−1^ at MBH-B. At the MOS and WIS stream average nitrate concentrations were significantly higher and ranged between 0.36 (± 0.02) and 0.44 (± 0.01) mmol L^−1^ (supplementary material, Table S1).

ArcGIS analyses of the digital elevation map of Bavaria, the stream network, and land cover maps provided land use proportions within the catchments upstream from each sampling point. The land use types in these catchment parts were classified into the following classes:

1. forest
2. grassland
3. agriculture
4. urban areas
5. water
6. natural to seminatural environments.

Of these classes only the first four were relevant for the analyses conducted in the parts of the catchments studied.

**Figure 4:**
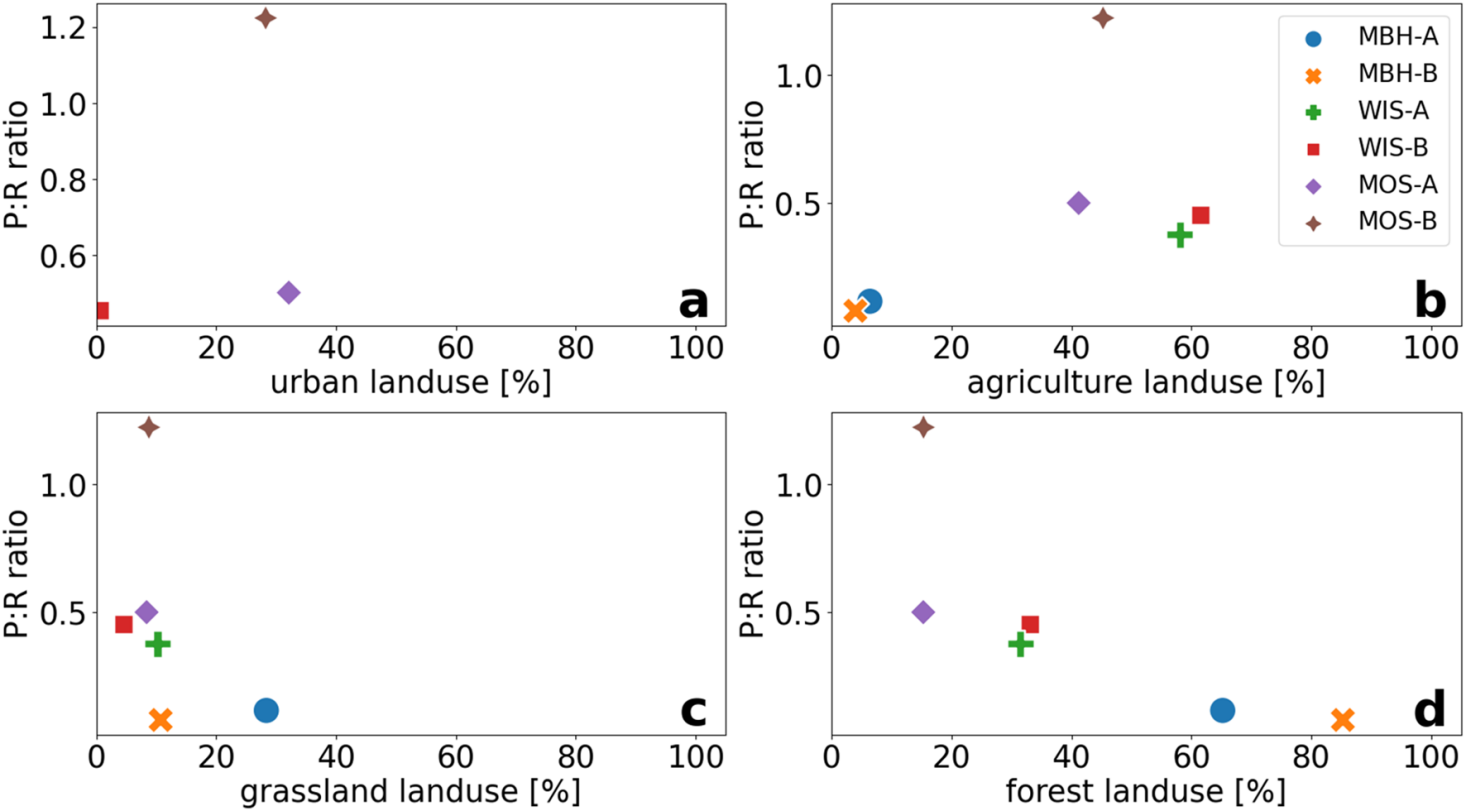
Land use type proportions in relation to modelled midnight-to-midnight photosynthesis to respiration (P:R) ratios within the catchments of the respective upstream (A) and downstream (B) sampling sites at the streams Mähringsbach (MBH), Wiesent (WIS) and Moosach (MOS). Shown land use types are urban (**a**), agriculture (**b**), grassland (**c**) and forest (**d**).

The part of the MBH stream catchment that influenced the two sampling sites is mainly composed of forests and grassland with a low proportion of agriculture (Table 5). In contrast, the analyzed parts of the WIS stream partial catchment showed more pronounced anthropogenic influences with approximately 60% agriculture as the most frequently encountered land use type and about 30% forests. The upstream parts of the sampling points along the MOS stream showed the highest anthropogenic influences with agricultural and urban land use. Natural land use types such as forests and grasslands were less important in these upstream parts of the catchment (Table 5 and Fig. 4).

**Table 5:** Land use classes and proportions in % in catchment parts upstream of respective sampling sites upstream (A) and further downstream (B) of the streams Mähringsbach (MBH), Wiesent (WIS) and Moosach (MOS).

Sampling site	Land use proportions					
	Forest (%)	Grassland (%)	Water (%)	Agriculture (%)	Urban (%)	Natural-seminatural (%)
MBH-A	65.2	28.4	–	6.4	–	–
MBH-B	85.3	10.7	–	4.0	–	–
WIS-A	31.5	10.3	–	58.2	–	–
WIS-B	33.1	4.6	–	61.6	0.6	–
MOS-A	15.3	8.4	1.6	41.2	32.1	1.4
MOS-B	15.3	8.8	1.3	45.3	28.3	1.1

When excluding G rates with the assumption that they are hardly influenced by land use, the evaluated land use types and calculated stream metabolisms from the PoRGy model showed good relationships with P:R ratios. In general, higher proportions of forests yielded lower P:R ratios (Fig. 4d), whereas elevated proportions of agriculture caused higher P:R ratios (Fig. 4b). Other land use types such as urban areas (Fig. 4a) or grasslands (Fig. 4c) only showed a small exposure in the catchments and had much lower influences on P:R ratios.

## Discussion

The overall performance of the PoRGy model with the application of DO saturations and additional stable isotopes was significantly better compared to tests when only DO concentrations were considered. This is because metabolic and G rates were better constrained especially at the less productive sites with higher contributions of G (Supplementary Material, Table S2). The literature describes various metabolism models that exclusively use DO concentrations^26,63–71^. Although most of them were able to model reasonable metabolic rates in streams with lower G rates, more turbulent flow conditions have been suggested to be more challenging. Here, the additional application of stable DO isotopes is able to better constrain P, R and G rates^63^. Working with well-constrained k values is essential to estimate and assess vulnerabilities of stream ecosystems in rapidly changing environments^27^. Such well-constrained k values might also help to define denitrification rates (via open-channel methods) and greenhouse gas flux rates (e.g. nitrous oxide). Such constrained k values could thus improve our general understanding of the contribution of terrestrial environments to global warming^72,73^.

Several stream metabolism studies applied *δ*^18^O_DO_ dynamics based on the same basic assumption of P and G as DO sources and R as a DO sink^29,30,67,74,75^. An advantage of the PoRGy model by Venkiteswaran et al.^24^ is that it was also translated into Matlab code that is freely available and easily applied.

In general, the metabolic P and R rates found in our study sites lie between the rates found in other stream systems, with lower P rates at forested study sites and higher at more anthropogenically influenced streams with higher proportions of agricultural and urban land use^76–79^.

The diel sampling sites at MBH stream represented the most pristine stream with high proportions of forest and grassland within the catchment and little agricultural activity (Fig. 4c,d). At both study sites of the MBH stream DO undersaturation and *δ*^18^O_DO_ values were above atmospheric equilibrium with a value of + 24.6‰^43,54^. This indicated a constant heterotrophic state of the stream (Fig. 3) that was also reflected by low P:R ratios (Table 4).

**Figure 5:**
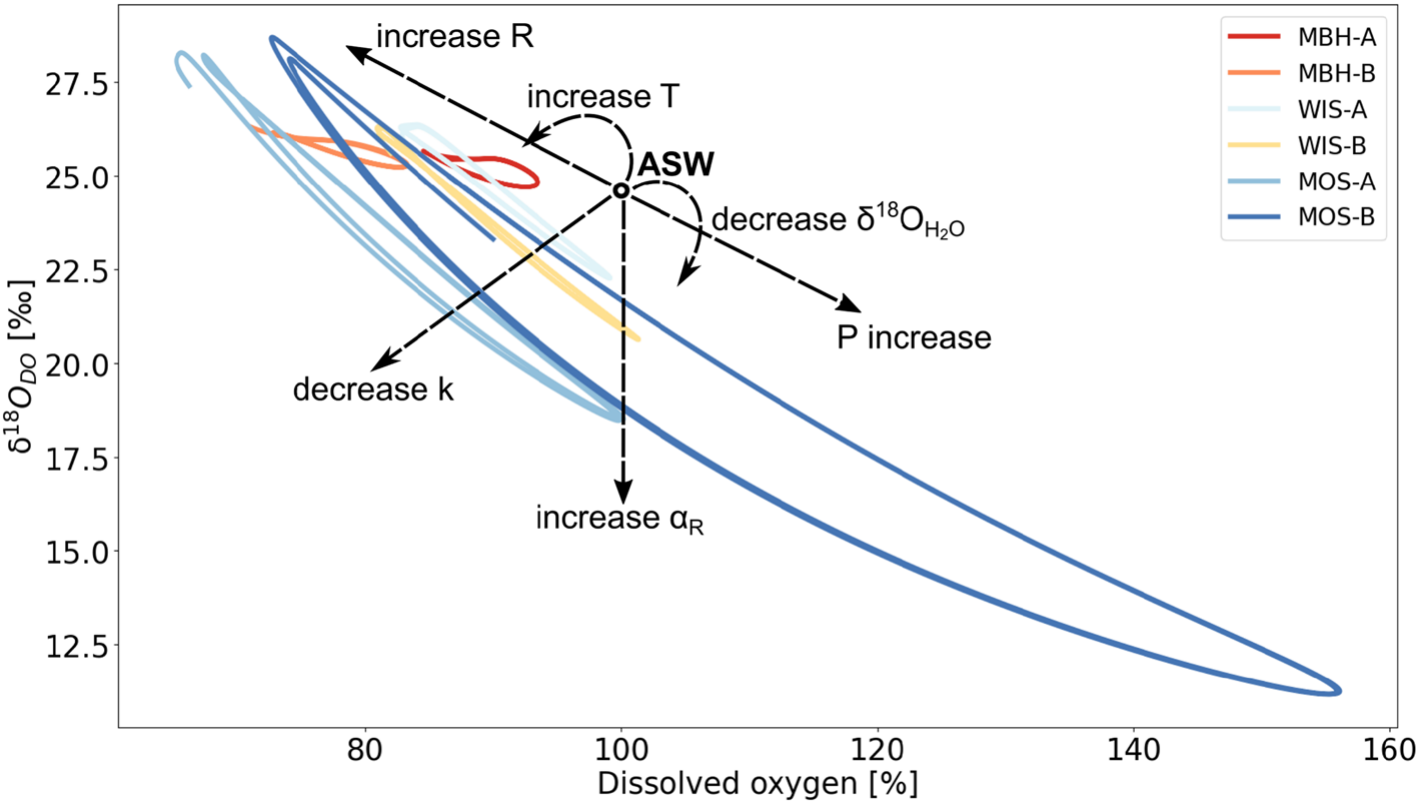
Summary of modelled diel cycles showing dissolved oxygen (DO) saturation versus DO stable isotopes (*δ*^18^O_DO_) of the streams Mähringsbach (MBH), Wiesent (WIS) and Moosach (MOS) with respective upstream (A) and downstream (B) sites. Processes related to position and range of day-night curves are photosynthesis (P), ecosystem respiration (R), respiration isotope fractionation factor (α_R_), gas exchange coefficient (k), stable water isotopes (*δ*^18^O_H2O_) and water temperature (T). The empty black circle represents air saturated water (ASW) at a DO saturation of 100% and δ^18^O_DO_ value of + 24.6‰^31^.

The upstream catchment part of site MBH-A had more than 60% forest coverage. It is also closest to the source. Therefore, increased shading by trees likely reduced solar radiation and resulted in typically low P rates. These low P rates were also observed in other forested headwater streams^37,80^. Simultaneously, high R rates are typically due to input of allochthonous material from high forest proportions in the catchment, which caused the constant DO undersaturation^37^. Among the streams, k was highest at the site MBH-A (Table 4). This likely resulted from turbulent flow conditions caused by stream bed roughness, shallow water depths and steeper stream slopes. These driving factors for k were also observed at other headwater streams^81,82^. Elevated k values that cause high G rates can in turn cause DO undersaturation due to R at night. Such combinations can generally dampen day-night amplitudes (Figs. 1 and 5). However, particularly in this shallow headwater stream section, estimates of k also caused uncertainties in model outputs because turbulence and the possibility of air bubble inclusion may be underestimated^81,83^. This might have been one of the reasons for the poorer model fit at this study site (Fig. 3). Additionally, effects of shading by trees are not considered in the model which only includes latitude, altitude and daytime specific solar influences for the calculation of P. Another factor that may have caused higher uncertainties is that smaller water volumes of the stream can become affected more easily by short-term changes in P, R and G due to altered nutrient concentration, discharge, water temperatures and exchange with the soil water and HZ. In contrast, inputs of significant amounts of groundwater seem to be less probable because the bedrock in this area had a low permeability in this study area. These uncertainties can result in untypical day-time DO and *δ*^18^O_DO_ patterns that were evident at study site MBH-A. These aspects render this site most challenging for modelling.

MBH-B was located outside the forested section. Therefore, less shading by vegetation may have enhanced photosynthetically active radiation (PAR) that in turn can trigger higher metabolic rates. However, minor effects on PAR could still have been caused by shrubs in the vicinity of the stream that may have caused partial shading of the stream water. The model yielded even lower metabolic rates with similar ratios when compared to the upstream sampling site (Table 4). However, the model fit was considerably better than at MBH-A. This implies that the higher metabolic rates observed at MBH-A may be due to model uncertainties and should be interpreted with caution. Nevertheless, the low modelled P:R ratios at MBH-B may also mark residual effects of the larger proportions of forests further upstream (Table 5, Fig. 4d). Additionally, nutrients such as nitrate and phosphate were very low at both study sites due to low agricultural land use proportions. This may have hampered photosynthetic DO production, even when PAR became higher at MBH-B.

The two study sites at the WIS stream were only 400 m apart from each other and therefore showed similar diel DO and *δ*^18^O_DO_ ranges (Table 3, Fig. 3). Both study sites only approached DO saturations of atmospheric equilibrium around the time of the solar peak around 13:00 h. The remainder of the day, and especially during the night, both study sites became undersaturated in DO. Here the model results indicated a heterotrophic state of the stream with a dominance of R that outcompeted rates of G and P.

Also note that sampling at the WIS stream was also influenced by alternating cloud cover with showers towards the end of the sampling event. These meteorological conditions may have reduced P as a result of reduced solar radiation by cloud shading while the rainfall could have increased turbidity in the water as a result of terrestrial sediment input from the surrounding area or from stream bed erosion^84,85^. Moreover, the precipitation events could have also increased R rates with more labile terrestrial carbon being washed into the stream. Subsequently, these inputs may increase heterotrophic respiration^64,86^. The higher proportion of agricultural land use found in the catchment of the WIS stream could enhance elevated inputs of nutrients which would increase P (Table 5). On the other hand, agriculturally worked soil could have caused more rapid increases in turbidity by enhancing mobilization of soil material. This would in turn have reduced P and may even outcompete effects of nutrient inputs^87,88^. Moreover, rainfall-related increased inputs of groundwater in the karstic region at the study sites during sampling may have affected P and R rates due to mixing^89^. These causes and effects could best be observed during the strong rainfall events at the end of the diel sampling, which showed a day-time atypical reduction of DO and an increase of *δ*^18^O_DO._ Both values indicate reduced P and increased R rates. This shows that unstable weather conditions can cause irregularities of diel patterns and render modelling of the stream sites more challenging. Despite such unstable weather conditions, metabolic processes were still more pronounced in the WIS stream than in the MBH stream. This was reflected by a larger diel range of DO and *δ*^18^O_DO_ values (Fig. 5). Due to the proximity of both study sites to its spring, the observed dominance of R rates may also reflect a residual signal from upstream DO-undersaturated spring water that was also measured by van Geldern et al.^90^.

Although P rates at the WIS stream were much smaller than their corresponding R rates, they still were 2 to 7-fold higher than those in the MBH stream (Table 4). Nonetheless, these DO values never reached significant oversaturation. Also, the *δ*^18^O_DO_ values were lower than + 24.6‰ during most of the day and only reached values higher than + 24.6‰ after sunset (Fig. 3). This shows the large effect of photosynthetic oxygen production with an average value of around − 9‰ via splitting of water molecules. This process then adds oxygen with a lower signal to the DO pool. This process seems to be important even in a R-dominated stream. A possible explanation for higher P rates can be inferred from a combination of PAR and nutrient availability. In contrast to the MBH stream with large proportions of forest in the catchment, the WIS stream catchment has more agricultural areas and grasslands (Table 5). This also implies that the upstream parts of the WIS stream are not fully shaded and only covered by smaller shrubs or few trees. This in turn allows higher PAR at the water surface. Additionally, at the WIS stream elevated nitrate concentrations were derived from the nitrate-rich spring and groundwater input from agriculturally influenced land use. This was combined with reduced nutrient attenuation potential of the karstic bedrock^90^. The elevated nutrient concentrations in combination with reduced shading likely fueled the photosynthetic activity during day time even in the proximity of the spring. This suggests that P rates may reach even higher values on a sunny day.

In this heterotrophic stream, G acted as an important parameter to counterbalance DO consumption at night. With stronger DO undersaturation G rates increased, until G and R rates became balanced during night. This balance helped to establish well-pronounced night plateaus of DO and *δ*^18^O_DO_ at both study sites (Fig. 3).

In contrast to the above, study sites of the MOS stream showed differing day-night patterns of DO and *δ*^18^O_DO_ and produced the highest metabolic variations among the three streams. The upstream study site, MOS-A, maintained heterotrophic conditions with DO undersaturation during night and most of the day with a low P:R ratio and a high R rate (Fig. 3, Table 4). Due to this constantly high oxygen demand, DO saturations were lowest at night when compared to the other streams. This undersaturation was mostly counterbalanced by G. Generally, k values were lowest at the MOS stream (Table 4). This may have been another reason for the low DO saturation plateau at night. Due to high R and low k, corresponding *δ*^18^O_DO_ values reached their highest values when compared to the other two streams (Fig. 5). DO values only approached saturation, when DO input by high P and G was large enough during the day. The elevated P rates at this study site caused input of photosynthetic oxygen during the day with its distinct isotope signal. This is best reflected in the rapid shift from respiration-dominated *δ*^18^O_DO_ values above those of atmospheric equilibrium at night in relation to photosynthetic signals below + 24.6‰ during the day (Fig. 3).

Compared to the upstream MOS study site DO saturations and *δ*^18^O_DO_ reached similarly high values during absence of light at MOS-B. This indicates that all DO consuming processes and G must have been on an equal level at both study sites. Generally, the downstream P rate was 2.6-fold higher when compared to the upstream study site MOS-A (Table 4). Therefore, this is the only study site, where P became the dominant part of the metabolism. This was also evident by a P:R ratio above 1. This active metabolism also caused more pronounced day-night curves with high P rates and DO oversaturation during the day and subsequently rapidly declining DO values during the night. Because R rates and k were similar at both study sites these elevated P rates were the main drivers of altered DO and *δ*^18^O_DO_ curves during daytime.

Among the three studied streams, MOS showed the strongest anthropogenic impact by urban and agricultural practices. The river consists of a complex channel system with the MOS main stream as the integral collector. Both land use types generally relate to increased nutrient inputs such as nitrate, high biochemical oxygen demand, increased water temperatures and reduced shading^91,92^. The lower productivity found at study site MOS-A compared to MOS-B could stem from lower PAR because of partial shading by shrubs and trees at the study location and the stream water further upstream. Although at study site MOS-B was also surrounded by larger trees, which caused shading, the further upstream section only had sparse vegetation covers. Therefore, high DO by P may have been caused by residual upstream inputs. However, also increased input of DO-depleted groundwater or exchanges of groundwater via the hyporheic zone in MOS-A could have dampened DO curves and may have decreased T.

## Conclusions

Diel measurements of DO saturations and associated *δ*^18^O_DO_ values in three contrasting streams within the same climate zone each showed distinct diel curves that correlated with proportions of various land use forms in their catchments, proximities to their springs and weather conditions. These factors can in turn affect nutrient concentrations and PAR due to shading. The PoRGy model successfully matched diel field data to estimate important P, R and G rates. Here, the additional application of stable DO isotopes substantially improved the model output by constraining metabolic rates and k values. This modelling approach also enabled a synoptic view of different metabolic states of the stream sections. Uncertainties of model outputs can be attributed to tree canopy, alternating weather conditions and groundwater input. These factors were not directly considered in the model but likely influenced its outcomes.

Notably, all sites (except for one) were predominantly undersaturated in DO and  confirmed heterotrophic states. This was marked by low P:R ratios. DO undersaturation combined with low productivity and elevated k values, caused atmospheric oxygen to become the dominant oxygen source in the MBH and the WIS stream. Stream sections with higher k values are considered more resilient to anthropogenic or climatic changes because elevated G serves as a reliable and constant oxygen input. In contrast, the two sites at the most anthropogenically influenced stream showed the highest metabolic DO turnover ranges. Here, larger DO amplitudes between upstream and downstream locations highlight effects of shading on the productivity of the stream during the day. Although estimated k values at these sites were lowest at the MOS stream, G was still sufficient to avoid severe DO depletion overnight. However, climatic changes with rising temperatures could further increase heterotrophy in the streams while lowering DO solubility and increasing respiration^20^.

The PoRGy model is a promising tool to determine ecological states of stream sections in an integral manner. It enables direct comparisons of the P, R and G rates. However, further testing should be performed at higher resolution and over longer time periods. This could be arranged by sampling via specialised autosamplers that isolate samples from atmospheric influences. Such higher frequency data would also help to increase the accuracy of the model. Moreover, model uncertainties could be improved by direct determinations of k and PAR.

Overall, our data provide robust early warning information for improved stream and river management to help mitigate effects of land use and climate change. Stream comparisons of this study yielded smaller differences within streams than comparison between streams. This seems to be mostly related to different proportions of land use. However, when comparing sites over the entire length of streams more pronounced differences may become obvious between sources and mouths of rivers.

The ecological importance of streams and rivers becomes increasingly recognized. In particular, human activities can severely affect water chemistry of river networks as integral reflectors of their catchments. Further applications of this technique at selected parts of rivers including tributaries and mouth sections would help evaluate entire catchments. In addition, diel changes of DO and its isotopes need to be further investigated in different seasons to establish better understanding of annual dynamics. Moreover, dynamic models that capture variable conditions such as high water stands or receding limbs of hydrographs, would improve the understanding of stream metabolism responses to a changing environment.

## Supplementary Information

Supplementary Tables.
