# Supplementary material for: Dissolved oxygen isotope modelling refines metabolic state estimates of stream ecosystems with different land use background
Source: Sci Rep. 2022 Jun 17;12:10204. doi: 10.1038/s41598-022-13219-9 (PMC9205993; doi:10.1038/s41598-022-13219-9)
Supplement: Supplementary file 1 — Supplementary Tables. [file 41598_2022_13219_MOESM1_ESM.pdf]

Supplementary material

Dissolved oxygen isotope modelling refines metabolic state estimates of stream ecosystems with different land use background

David R. Piatka[1, 5 *], Jason J. Venkiteswaran[2], Bhumika Uniyal[3], Robin Kaule[4], Benjamin Gilfedder[4], Johannes A.C. Barth[1]

*corresponding author

[1]Department of Geography and Geosciences, GeoZentrum Nordbayern, Friedrich-Alexander-Universität Erlangen-Nürnberg (FAU), Schlossgarten 5, D-91054 Erlangen, Germany

[2]Department of Geography and Environmental Studies, Wilfrid Laurier University, 75 University Avenue West, Waterloo, ON, N2L 3C5, Canada

[3]Professorship of Ecological Services, Bayreuth Center of Ecology and Environmental Research (BayCEER), University of Bayreuth, Universitaetsstr. 30, 95447 Bayreuth, Germany

[4]Limnological Research Station, BayCEER, Department of Hydrology, University of Bayreuth, 95440 Bayreuth, Germany

[5]Current address: Karlsruhe Institute of Technology, Institute of Meteorology and Climate Research (IMK-IFU), Kreuzeckbahnstr. 19, 82467 Garmisch-Partenkirchen, Germany

Table S1. Summary of measured dissolved oxygen (DO), water temperatures, nitrate, phosphate, , and stable oxygen isotopes of DO ($\delta^{18}O_{DO}$) and water ($\delta^{18}O_{H2O}$) at the streams Moosach (MOS), Mähringsbach (MBH) and Wiesent (WIS) with respective upstream (A) and downstream (B) sampling sites.

Date [mm/dd/yy]	Time [hh:mm]	Study site	DO [mg L^{-1}]	DO [%]	Temperature [°C]	Nitrate [mmol L^{-1}]	Phosphate [mmol L^{-1}]	$\delta^{18}O_{H2O}$ [‰]	Mean $\delta^{18}O_{DO}$ [‰]
07/16/2019	10:00	MOS-A	8.0	84.3	13.9	0.41	-	-9.4	22.4
07/16/2019	12:00	MOS-A	8.9	92.6	14.3	-	-	-	20.4
07/16/2019	14:00	MOS-A	9.2	96.2	14.9	0.41	-	-	19.1
07/16/2019	16:00	MOS-A	9.3	99.7	15.9	-	-	-	18.9
07/16/2019	18:00	MOS-A	8.8	95.5	16.5	0.41	-	-	19.6
07/16/2019	20:00	MOS-A	7.9	84.7	16.3	-	-	-	22.5
07/16/2019	22:00	MOS-A	6.9	73.0	15.8	0.41	-	-9.4	25.7
07/17/2019	00:00	MOS-A	6.5	67.4	14.9	-	-	-	27.4
07/17/2019	04:00	MOS-A	6.7	67.5	13.3	0.41	-	-	27.9
07/17/2019	06:00	MOS-A	7.0	70.2	12.9	0.41	-	-	27.9
07/17/2019	08:00	MOS-A	7.5	74.6	13.0	-	-	-	25.5
07/17/2019	10:00	MOS-A	8.6	87.1	13.4	0.41	-	-9.4	22.0
07/17/2019	12:00	MOS-A	8.8	91.2	14.1	-	-	-	19.8
07/17/2019	14:00	MOS-A	9.2	97.3	15.3	0.41	-	-	18.8
07/16/2019	10:00	MOS-B	11.7	119.2	14.4	0.39	-	-9.5	16.5
07/16/2019	12:00	MOS-B	13.0	135.8	15.2	-	-	-	14.8
07/16/2019	14:00	MOS-B	13.9	147.6	16.1	0.39	-	-	13.4
07/16/2019	16:00	MOS-B	13.9	149.8	16.6	-	-	-	13.2
07/16/2019	18:00	MOS-B	13.3	144.7	16.9	0.39	-	-	13.6
07/16/2019	20:00	MOS-B	11.6	125.5	16.6	-	-	-	16.5
07/16/2019	22:00	MOS-B	9.2	98.1	16.3	0.39	-	-9.5	21.0
07/17/2019	00:00	MOS-B	8.6	88.4	14.6	-	-	-	23.3
07/17/2019	02:00	MOS-B	6.8	71.3	15.3	0.39	-	-	26.8
07/17/2019	04:00	MOS-B	6.4	65.9	14.8	-	-	-	28.1
07/17/2019	06:00	MOS-B	6.5	66.4	14.3	0.38	-	-	27.7
07/17/2019	08:00	MOS-B	8.2	84.9	14.6	-	-	-	21.8
07/17/2019	10:00	MOS-B	11.5	118.3	14.5	0.38	-	-9.5	15.8
07/17/2019	12:00	MOS-B	13.0	136.5	15.3	-	-	-	14.1
07/17/2019	14:00	MOS-B	14.1	151.2	16.2	0.39	-	-	12.7
07/24/2019	10:00	MBH-A	9.2	93.3	13.7	0.08	-	-9.4	25.1
07/24/2019	12:00	MBH-A	8.8	93.0	15.4	-	-	-	-
07/24/2019	14:00	MBH-A	8.0	91.1	18.5	0.08	-	-	24.8
07/24/2019	16:00	MBH-A	7.4	86.2	19.7	-	-	-	25.1
07/24/2019	18:00	MBH-A	7.2	84.1	19.9	0.07	0.02	-	25.4
07/24/2019	20:00	MBH-A	7.2	83.1	19.3	-	-	-	25.6
07/24/2019	22:00	MBH-A	7.5	83.5	18.0	0.06	-	-9.2	25.6
07/25/2019	00:00	MBH-A	8.2	89.3	16.5	-	-	-	25.4
07/25/2019	02:00	MBH-A	8.7	91.0	14.8	0.05	-	-	25.2
07/25/2019	04:00	MBH-A	9.0	92.1	14.0	-	-	-	25.2
07/25/2019	06:00	MBH-A	9.1	91.6	13.1	0.06	-	-	25.1
07/25/2019	08:00	MBH-A	9.2	94.4	13.9	0.07	-	-	25.0
07/25/2019	10:00	MBH-A	9.0	93.3	14.2	0.07	-	-9.4	24.9
07/25/2019	12:00	MBH-A	8.6	92.9	16.2	-	-	-	24.8
07/25/2019	14:00	MBH-A	7.8	96.4	19.4	0.07	-	-	24.8
07/25/2019	16:00	MBH-A	7.2	84.5	20.4	-	-	-	25.3
07/24/2019	10:00	MBH-B	7.7	83.9	16.8	0.00	-	-7.9	25.5
07/24/2019	12:00	MBH-B	7.6	85.2	17.9	-	-	-	25.3
07/24/2019	14:00	MBH-B	7.3	83.9	19.5	0.08	-	-	25.2
07/24/2019	16:00	MBH-B	6.8	81.6	21.1	-	-	-	25.3
07/24/2019	18:00	MBH-B	6.3	76.9	22.4	0.08	-	-	25.6
07/24/2019	20:00	MBH-B	6.0	74.3	22.6	-	-	-	26.0
07/24/2019	22:00	MBH-B	6.0	72.0	21.7	0.08	-	-7.8	26.2
07/25/2019	00:00	MBH-B	6.3	73.7	20.2	-	-	-	26.1
07/25/2019	02:00	MBH-B	6.6	75.1	18.9	0.07	-	-	26.0
07/25/2019	04:00	MBH-B	6.8	76.2	17.9	-	-	-	25.9
07/25/2019	06:00	MBH-B	7.1	77.6	17.2	0.07	-	-	25.8
07/25/2019	08:00	MBH-B	7.2	79.2	17.2	-	-	-	25.6
07/25/2019	10:00	MBH-B	7.3	81.5	17.9	0.28	-	-7.8	25.5
07/25/2019	12:00	MBH-B	7.2	82.2	19.2	-	-	-	25.3
07/25/2019	14:00	MBH-B	6.8	79.4	20.2	0.08	-	-	25.6
08/06/2019	10:00	WIS-A	10.2	99.5	12.3	-	-	-9.4	23.2
08/06/2019	12:00	WIS-A	10.1	100.6	13.1	-	-	-	22.7

Date [mm/dd/yy]	Time [hh:mm]	Study site	DO [mg L-1]	DO [%]	Temperature [°C]	Nitrate [mmol L^{-1}]	Phosphate [mmol L^{-1}]	$\delta^{18}O_{H2O}$ [‰]	Mean $\delta^{18}O_{DO}$ [‰]
08/06/2019	14:00	WIS-A	9.5	95.3	13.7	0.38	0.00	-	23.6
08/06/2019	16:00	WIS-A	9.4	97.0	14.8	-	-	-	22.9
08/06/2019	18:00	WIS-A	9.2	95.6	15.1	0.36	0.00	-	23.2
08/06/2019	20:00	WIS-A	8.6	89.2	14.9	-	-	-	24.5
08/06/2019	22:00	WIS-A	8.3	84.8	14.3	0.35	0.00	-9.4	25.6
08/07/2019	00:00	WIS-A	8.4	84.7	13.7	-	-	-	25.7
08/07/2019	02:00	WIS-A	8.4	84.0	13.0	0.35	0.00	-	25.8
08/07/2019	04:00	WIS-A	8.7	85.4	12.2	-	-	-	25.8
08/07/2019	06:00	WIS-A	8.9	86.6	11.8	0.35	0.00	-	25.9
08/07/2019	08:00	WIS-A	9.1	88.8	12.0	-	-	-	24.8
08/07/2019	10:00	WIS-A	9.3	91.4	12.3	0.35	0.00	-8.9	24.2
08/07/2019	12:00	WIS-A	8.9	88.9	13.3	0.34	0.00	-	22.9
08/07/2019	14:00	WIS-A	9.2	92.6	13.6	0.41	0.00	-	24.9
08/06/2019	10:00	WIS-B	10.2	96.4	10.7	0.45	0.00	-9.5	22.3
08/06/2019	12:00	WIS-B	10.6	102.3	11.6	-	-	-	21.1
08/06/2019	14:00	WIS-B	10.1	97.6	11.6	0.45	0.00	-	22.4
08/06/2019	16:00	WIS-B	10.3	100.9	12.0	-	-	-	21.1
08/06/2019	18:00	WIS-B	9.9	97.6	12.5	0.44	0.00	-	21.8
08/06/2019	20:00	WIS-B	9.1	88.8	12.2	-	-	-	23.6
08/06/2019	22:00	WIS-B	8.5	82.2	11.7	0.44	0.00	-	25.3
08/07/2019	00:00	WIS-B	8.6	84.1	11.5	-	-	-9.4	25.6
08/07/2019	02:00	WIS-B	8.4	80.7	11.2	0.44	0.00	-	25.6
08/07/2019	04:00	WIS-B	8.5	80.9	10.9	-	-	-	25.6
08/07/2019	06:00	WIS-B	8.6	81.8	10.7	0.44	0.00	-	25.6
08/07/2019	08:00	WIS-B	8.9	84.9	10.5	-	-	-	24.8
08/07/2019	10:00	WIS-B	9.4	89.6	10.8	0.43	0.00	-9.1	23.6
08/07/2019	12:00	WIS-B	10.3	99.9	11.8	-	-	-	21.3
08/07/2019	14:00	WIS-B	9.6	93.3	11.6	0.44	0.00	-	22.8

Table S2. Overview of modelled midnight-to-midnight photosynthesis (P), ecosystem respiration (R) and gross gas exchange (G*) rates and ratios, and gas exchange (G) coefficients (k) with goodness of fit (r^2) of the PoRGy model only based on dissolved oxygen concentrations (DO) at the streams Mähringsbach (MBH), Wiesent (WIS) and Moosach (MOS) with respective upstream (A) and downstream (B) sites.

	**Midnight-to-midnight**							
**Study site**	**Goodness of fit (r^2)**	**P rate [mg m^{-2} h^{-1}]**	**R rate [mg m^{-2} h^{-1}]**	**G* rate [mg m^{-2} h^{-1}]**	**P : R ratio**	**P : G ratio**	**P : R : G ratio**	**k [m h^{-1}]**
MBH-A	0.52	180	701	515	0.3	0.4	0.4: 1.4:1	0.50
MBH-B	0.89	371	306	270	0.1	0.1	0.1:1.1:1	0.14
WIS-A	0.64	101	255	152	0.4	0.7	0.7:1.7:1	0.15
WIS-B	0.85	146	313	171	0.5	0.9	0.9:1.8:1	0.14
MOS-A	0.97	168	342	180	0.5	0.9	0.9:1.9:1	0.09
MOS-B	0.97	456	386	242	1.2	1.9	1.9:1.6:1	0.10
